# DGKδ triggers endoplasmic reticulum release of IFT88-containing vesicles destined for the assembly of primary cilia

**DOI:** 10.1038/s41598-017-05680-8

**Published:** 2017-07-13

**Authors:** Jie Ding, Lei Shao, Yixing Yao, Xin Tong, Huaize Liu, Shen Yue, Lu Xie, Steven Y. Cheng

**Affiliations:** Department of Developmental Genetics, School of Basic Medical Sciences, Nanjing Medical University, 101 Longmian Avenue, Nanjing, Jiangsu 211166 China

## Abstract

The morphogenic factor Sonic hedgehog (Shh) signals through the primary cilium, which relies on intraflagellar transport to maintain its structural integrity and function. However, the process by which protein and lipid cargos are delivered to the primary cilium from their sites of synthesis still remains poorly characterized. Here, we report that diacylglycerol kinase δ (DGKδ), a residential lipid kinase in the endoplasmic reticulum, triggers the release of IFT88-containing vesicles from the ER exit sites (ERES), thereby setting forth their movement to the primary cilium. Encoded by the gene whose mutations originally implicated the primary cilium as the venue of Shh signaling, IFT88 is known to be part of the complex B that drives the anterograde transport within cilia. We show that IFT88 interacts with DGKδ, and is associated with COPII-coated vesicles at the ERES. Using a combination of RNAi silencing and gene knockout strategies, we further show that DGKδ is required for supporting Shh signaling both *in vitro* and *in vivo*, demonstrating the physiological significance of this regulation.

## Introduction

The Hedgehog morphogenic pathway plays conserved roles in organizing tissue pattern formation and cell fate determination during the development of a diverse array of organisms^1, 2^. In vertebrates, signaling by Sonic hedgehog (Shh) occurs through the primary cilium^3, 4^, a flagellum-like structure on the surface of interphase cells with properties akin to a signaling center^5, 6^. The current paradigm entails ligand-induced movements of receptor Patched1 (Ptch1) out of and membrane-bound signal transducer Smoothened (Smo) into the primary cilium^7–9^. Other cytosolic components of the pathway, including downstream transcriptional factors Gli1, 2, 3 and Gli-binding protein, Suppressor of fused (Sufu), also traverse into the primary cilium upon ligand induction^10–12^. Trafficking of these diverse types of proteins is orchestrated by intraflagellar transport (IFT) complexes and motor proteins^13, 14^; however, its precise mechanism remains to be determined.

The primary cilium was originally implicated as the venue of Shh signaling through characterization of two induced mouse mutants, *wimple* and *flexo/Polaris/Tg737*, which encode IFT172 and IFT88^3^, respectively, of a large multimeric complex that drives the anterograde ciliary movement along the microtubule-based axoneme^15^. Mouse mutants with different IFT88 lesions exhibit a wide range of phenotypes from polycystic kidney disease and retinal degeneration to abnormal brain morphology and preaxial polydactyly associated with disruption of Shh signaling^16, 17^. Mechanistically, IFT88 is essential for the assembly of the primary cilium, and the maintenance of its dynamic structural integrity^3, 17, 18^. Because no bio-synthetic machinery is present in cilia, all proteins must be imported into cilium by consecutive IFT movements across a basal barrier that effectively seals the primary cilium into a membrane-enclosed organelle^6, 19^. The sequence of many IFT proteins shares a common ancestry with vesicle coats of COPI, COPII and clathrin vesicles^20, 21^, which suggests that IFT proteins may also play a role in vesicular transport. This notion is supported by recent evidence implicating IFT20, IFT88 as well as IFT57 in exocytosis to facilitate the formation of and direct T-cell receptor targeting to immune synapses in T lymphocytes^22^. Moreover, a recent attempt at understanding causes of the Usher syndrome, a hereditary combined hearing and vision loss, identified IFT88 at the endoplasmic reticulum (ER)^23^, further lending evidence to a likely role in trafficking outside the primary cilium.

Here, we report a novel IFT88-binding protein diacylglycerol kinase δ (DGKδ), identified through affinity purification and mass spectrum analysis. A member of the DGK family lipid kinases that catalyze the conversion of diacylglycerol to phosphatidic acid^24^, DGKδ is a residential ER protein^25^. We show that IFT88 is associated with COPII-coated vesicles and it interacts with DGKδ at the ER exit site (ERES). The kinase activity of DGKδ is required for triggering the release of IFT88-containing COPII vesicles, thereby setting forth their movement toward the primary cilium. This process is essential to the structural and functional integrity of the primary cilium, henceforth Shh signaling.

## Results

### IFT88 is associated with ER and ER-derived COPII vesicles

**Figure 1:**
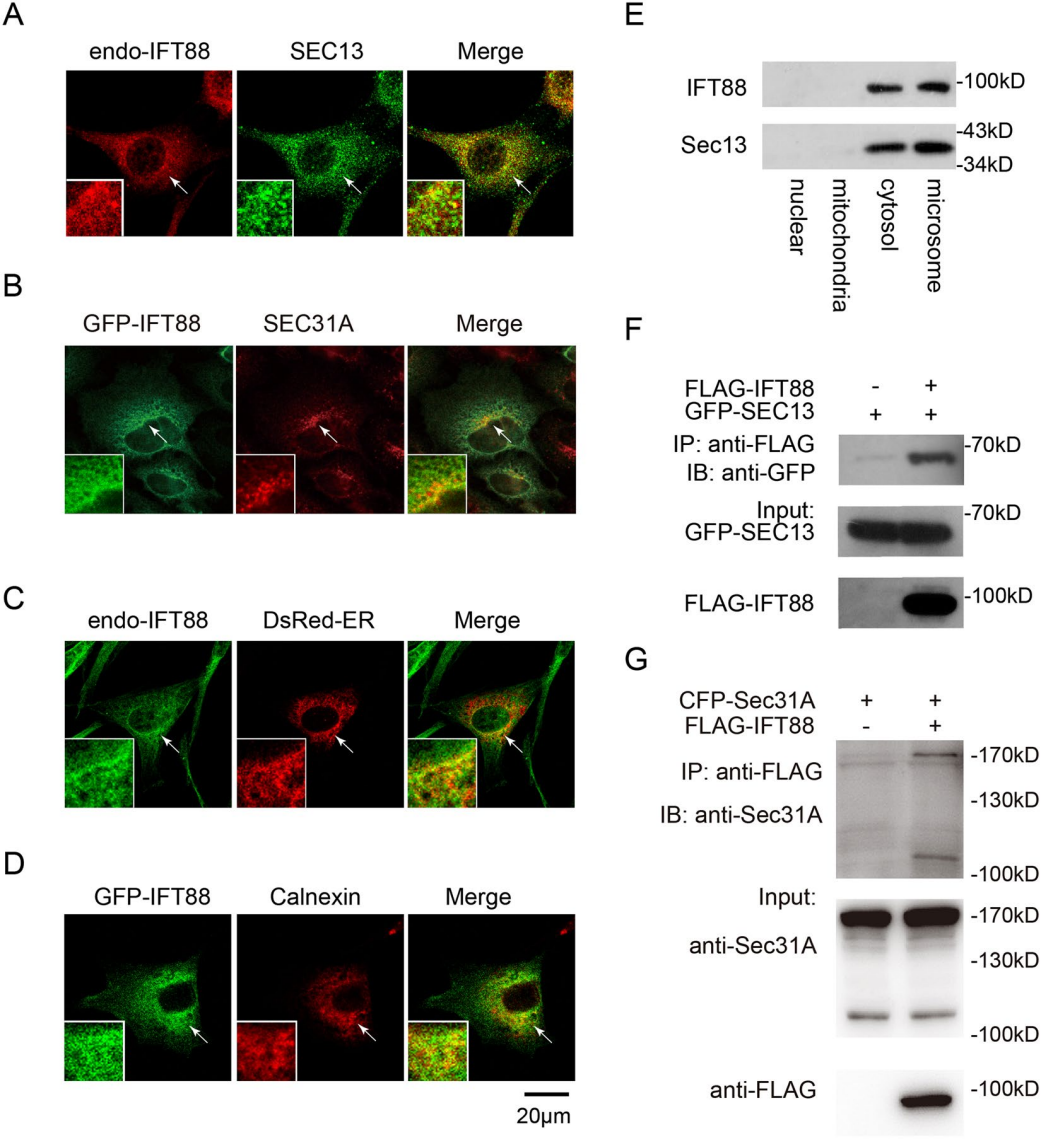
IFT88 is a component of ER-derived COPII coated vesicles. (**A**) Representative immunofluorescence staining images of endogenous IFT88 and SEC13 in the perinuclear region of NIH3T3 cells. The Manders Overlap Coefficient (MOC) between IFT88 and SEC13 is 0.72 ± 0.093. (**B**) Representative immunofluorescence confocal images showing colocalization of exogenously expressed GFP-IFT88 with endogenous Sec31A. MOC, 0.61 ± 0.03. (**C** and **D**) Representative confocal images showing colocalization of endogenous IFT88 with exogenously expressed ER marker DsRed-ER or exogenously expressed GFP-IFT88 with endo-Calnexin. Arrows mark the positions of the insets. MOC, 0.55 ± 0.11 in (**C**) and 0.68 ± 0.085 in (**D**), respectively. (**E**) Western analysis of IFT88 and SEC13 present in four differential centrifugation fractions in NIH3T3 cells. (**F** and **G**) Co-immunoprecipitation of exogenously expressed FLAG-IFT88 and GFP-Sec13 (**F**) or FLAG-IFT88 and CFP-Sec31A (**G**) in 293T cells. The cell lysates were precipitated with anti-FLAG M2 affinity gel and probed with anti-GFP or anti-Sec31A antibody for Western blot. The statistics analysis was computed based on data from at least 3 independent experiments.

To gain a global insight of the intracellular movement of IFT88, we conducted a survey of its localization in subcellular organelles in NIH3T3 cells using immunofluorescence microscopy. In addition to the primary cilium, the IFT88 staining extensively overlapped with that of SEC13 and SEC31A, both of which are part of the COPII-coated vesicles (Figs 1A,B, and S1A). The presence of IFT88 in the ER was also corroborated by the colocalization of endogenous IFT88 with the ER markers, DsRed-ER and Calnexin, as determined by immunofluorescence imaging (Fig. 1C and D), but the IFT88 staining did not overlap with markers for the Golgi apparatus (CFP-Gal, CFP-TGN38), endosomes (Lamp1, Rab5, Rab7) or Clathrin-coated vesicles (antibody against Clathrin heavy chain) (sFig. S1B–G). Biochemical fractionation experiments in NIH3T3 cells also showed association of IFT88 with Sec13^+^ microsomes but not mitochondria (Fig. 1E), thus corroborating the association of IFT88 with the ER and COPII vesicles. Finally, co-IP experiments using exogenously expressed FLAG-IFT88, GFP-Sec13, and CFP-SEC31A showed a physical interaction between IFT88 and the latter two proteins (Fig. 1F and G). Taken together, our data demonstrate that IFT88 is present in the ER and COPII vesicles and implicate IFT88 in transporting cargos from the ER to cilia by COPII vesicles.

### Identification of DGKδ as an interacting partner of IFT88

**Figure 2:**
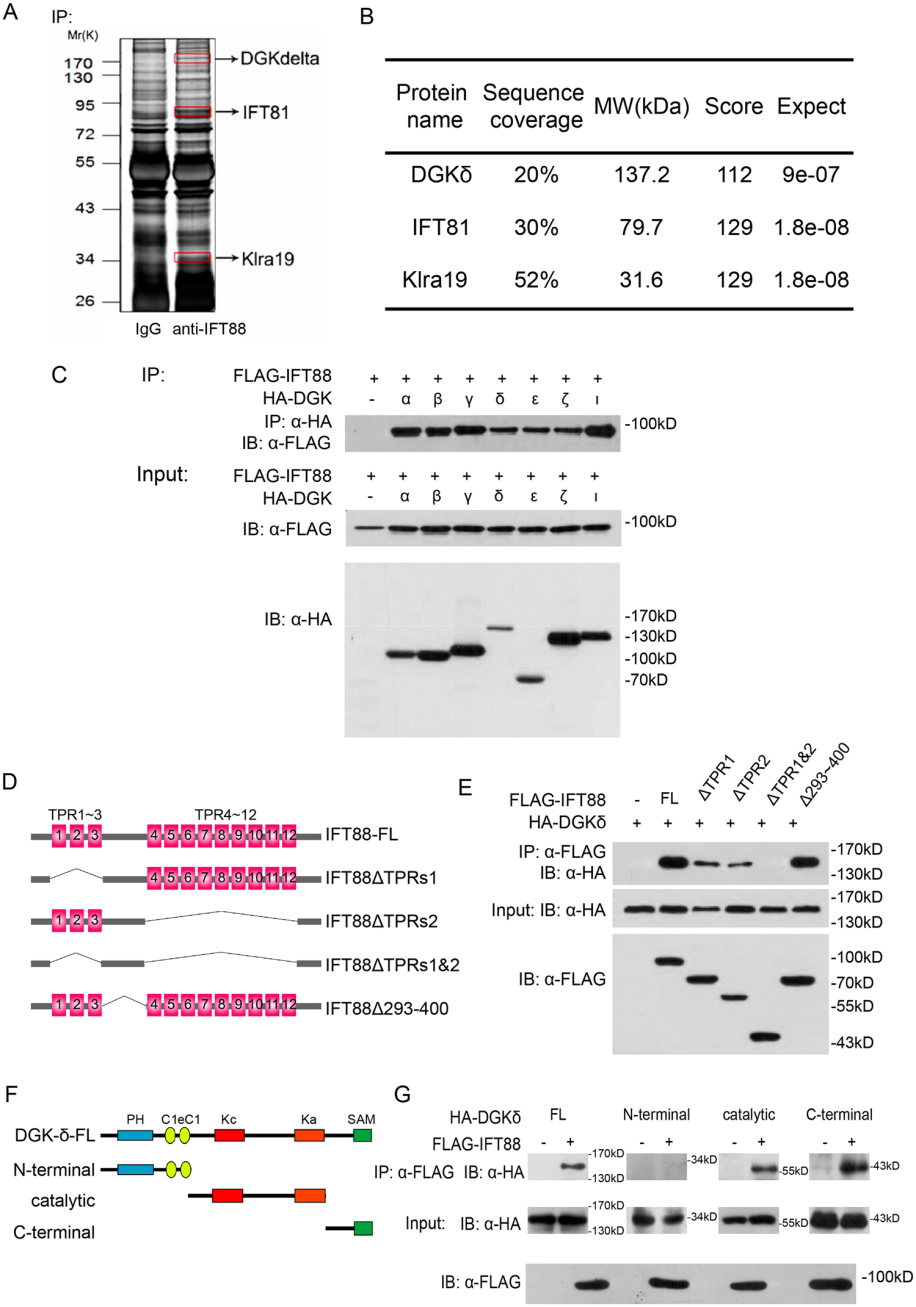
Identification of DGKδ as a specific IFT88 interacting protein. (**A**) Silver staining of proteins immunoprecipitated with anti-IFT88 or normal IgG control from NIH3T3 cell lysates and resolved by PAGE. 3 unique bands enriched in the anti-IFT88 lane were excised and identified by mass spectrometry. (**B**) Results from mass spectrometry analysis showing the identity of 3 bands to be: DGKδ, IFT81 and Klra19, respectively. (**C**) IP-Western analysis of exogenously expressed FLAG-tagged IFT88 and HA-tagged DGK isoforms in 293T cells. Top panel, anti-FLAG blotting after anti-HA immunoprecipitation. Total controls are shown in the bottom two panels. (**D**) Schematic representation of IFT88 domain structure and deletion constructs. (**E**) IP-Western analysis of the interaction between DGKδ and various IFT88 constructs. Total controls are shown in the bottom two panels. (**F**) Schematic representation of DGKδ domain structure and deletion constructs. (**G**) IP-Western analysis of the interaction between IFT88 and various DGKδ constructs.

To further explore the role and regulation of IFT88 in transporting ER cargos, we sought new IFT88 interacting proteins by immunoprecipitation and mass spectrometry identification in NIH3T3 cell lysates (Fig. 2A and B). Comparing to non-specific proteins that precipitated with the IgG control, at least 3 were uniquely enriched by anti-IFT88, as shown in the silver-stained electrophoresis lanes; these 3 proteins were identified as DGKδ, IFT81, and Klra19, respectively (Fig. 2A and B). Since IFT81 is a known component of the IFT-B complex^26^, its presence validated our approach. However, we were intrigued by DGKδ, an ER-resident lipid kinase and a key enzyme in the phospholipid biosynthesis pathway responsible for converting diacylglycerol to phosphatidic acid^25^, given the distinct phospholipid composition of the primary cilium membrane^27^. Moreover, mice deficient in DGKδ were born with open eyelids at birth^28^, a phenotype that could result from defects in primary cilium signaling^29^. For these reasons, DGKδ was chosen for further characterization.

The family of diacylglycerol kinases contains 10 isoforms^30^. Co-IP experiments revealed that IFT88 binds to all 7 DGK isoforms that we analyzed (Fig. 2C). The primary sequence of IFT88 consists of 12 tetratricopeptide repeats (TPRs) arranged in two asymmetric clusters^31^, and deletion mapping indicated that these TPR repeats likely mediate the interaction with DGKδ (Fig. 2D and E). On the other hand, the catalytic and C-terminal domains of DGKδ likely mediate the interaction with IFT88, whereas the N-terminal domain does not bind IFT88 (Fig. 2F and G). This result is consistent with the fact that all DGK isoforms tested bind IFT88 (Fig. 2C), since the catalytic domain is a structural element common to all members of the DGK family^24^.

### IFT88 and DGKδ are colocalized at the ER exit site

**Figure 3:**
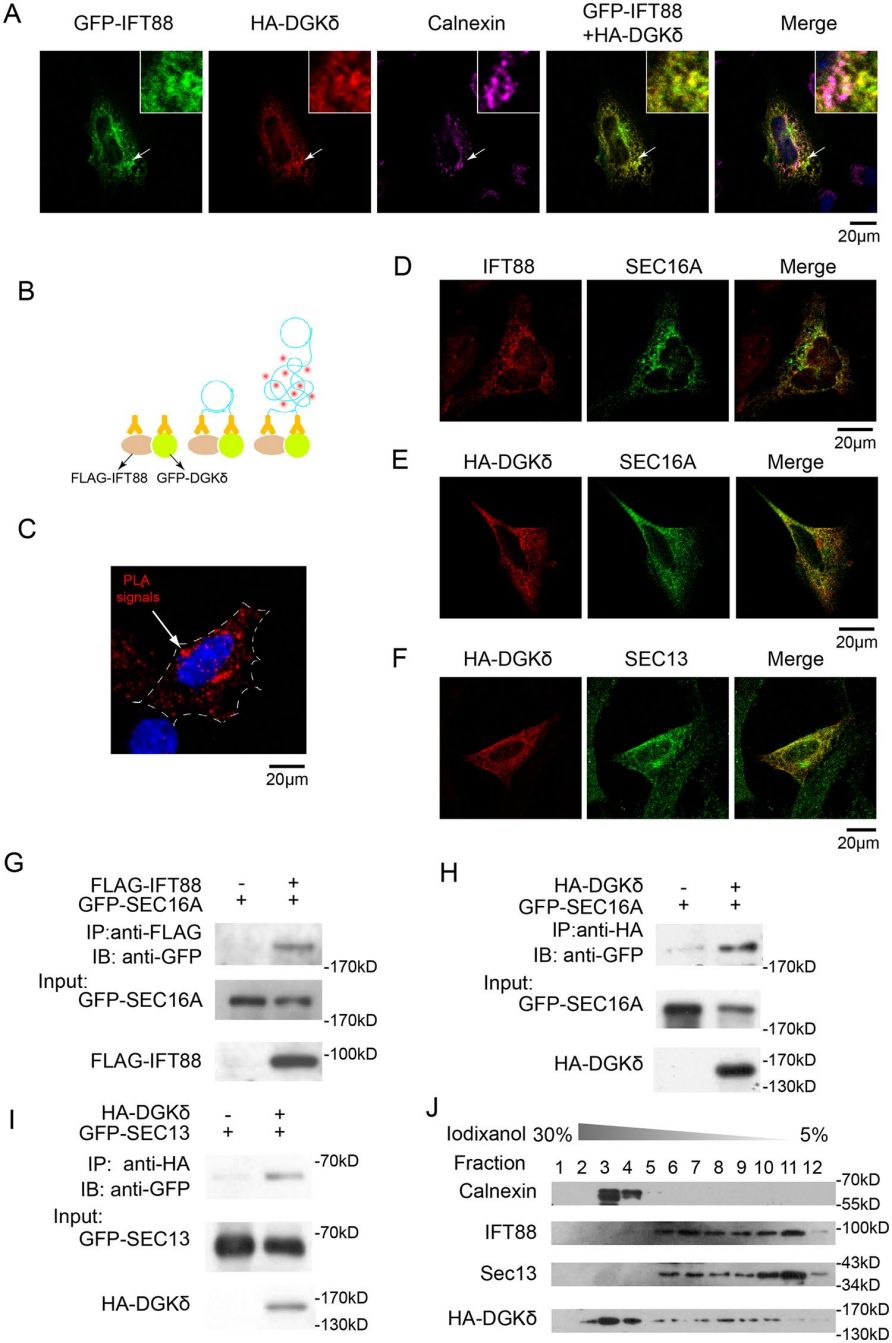
IFT88 is associated with COPII vesicles at the ER exit sites. (**A**) Representative immunofluorescence images of exogenously expressed GFP-IFT88, HA-DGKδ as well as endogenous calnexin in NIH3T3 cells. Arrows mark the positions of insets. MOC between IFT88 and DGKδ: 0.89 ± 0.065. (**B**) Schematic diagram and (**C**) a representative image of results from the proximity ligation assay showing that exogenously expressed FLAG-IFT88 and pEGFP-DGKδ are colocalized in the peri-nuclear region in NIH3T3 cells (the red signals adjacent came from another transfected cell). (**D–F**) Representative immunofluorescence images showing double staining of GFP-SEC16A and IFT88 (**D**), HA-DGKδ and GFP-SEC16A (**E**), as well as HA-DGKδ and SEC13 (**F**), respectively, in NIH3T3 cells. MOC: 0.68 ± 0.053 in (**D**), 0.81 ± 0.042 in (**E**), 0.63 ± 0.066 in (**F**). (**G**) IP-Western analysis of the interaction between exogenously expressed FLAG-IFT88 and GFP-SEC16A, (**H**) HA-DGKδ and GFP-SEC16A, as well as (**I**) HA-DGKδ and GFP-SEC13 in HEK293T cells. (**J**) Iodixanol density gradient sedimentation experiments in HEK293T cells, in which samples from 12 fractions were analyzed by Western blot for calnexin, IFT88, SEC13, and exogenously expressed HA-DGKδ. These experiments were reproduced in at least 3 independent experiments.

Having confirmed the interaction between IFT88 and DGKδ, we moved on to ascertain if and where the two proteins coexist in a complex at the ER. Confocal imaging showed that exogenously expressed HA-tagged DGKδ and GFP-tagged IFT88 were primarily co-localized in the perinuclear region marked by an ER marker Calnexin in NIH3T3 cells (Fig. 3A), but not at primary cilia (sFig. S2). The colocalization between IFT88 and DGKδ was further demonstrated by the proximity ligation assay (PLA), which also showed that the IFT88 and DGKδ complex was primarily present in the perinuclear region (Fig. 3B and C).

The COPII-coated vesicles are known to originate from the ER^32^. Since our data indicated that IFT88 is a component of the COPII vesicles, the interaction with IFT88 implies that DGKδ may participate in the regulation of COPII vesicle assembly or the release of COPII vesicles from the ER exit site (ERES) before they embark on secretory pathways. Indeed, immunofluorescence microscopy in transiently transfected NIH3T3 cells showed that both IFT88 and HA-DGKδ were co-localized with EGFP-SEC16A, which is a well-recognized marker for the ERES^33^ (Fig. 3D and E). Moreover, HA-DGKδ was also found co-localized with GFP-SEC13 (Fig. 3F), the marker of COPII-coated vesicle. Co-IP experiments confirmed the physical interaction of SEC16A to IFT88 and DGKδ, respectively (Fig. 3G and H), as well as SEC13 to DGKδ (Fig. 3I). Interestingly, experiments using equilibrium centrifugation through the iodixanol density gradient showed that DGKδ co-sedimented primarily in the heavy fractions labeled by the ER marker Calnexin, while IFT88 and SEC13 were mostly distributed in the lighter fractions (Fig. 3J). Taken together, our results indicated that while the majority of IFT88 likely exists in the free moving COPII vesicles, a fraction of which is associated with DGKδ in the ER at the ERES. These data suggest that DGKδ likely plays a role in the assembly of the IFT88-containing COPII vesicles or their release from the ER.

### DGKδ promotes the release of COPII vesicle from the ER

**Figure 4:**
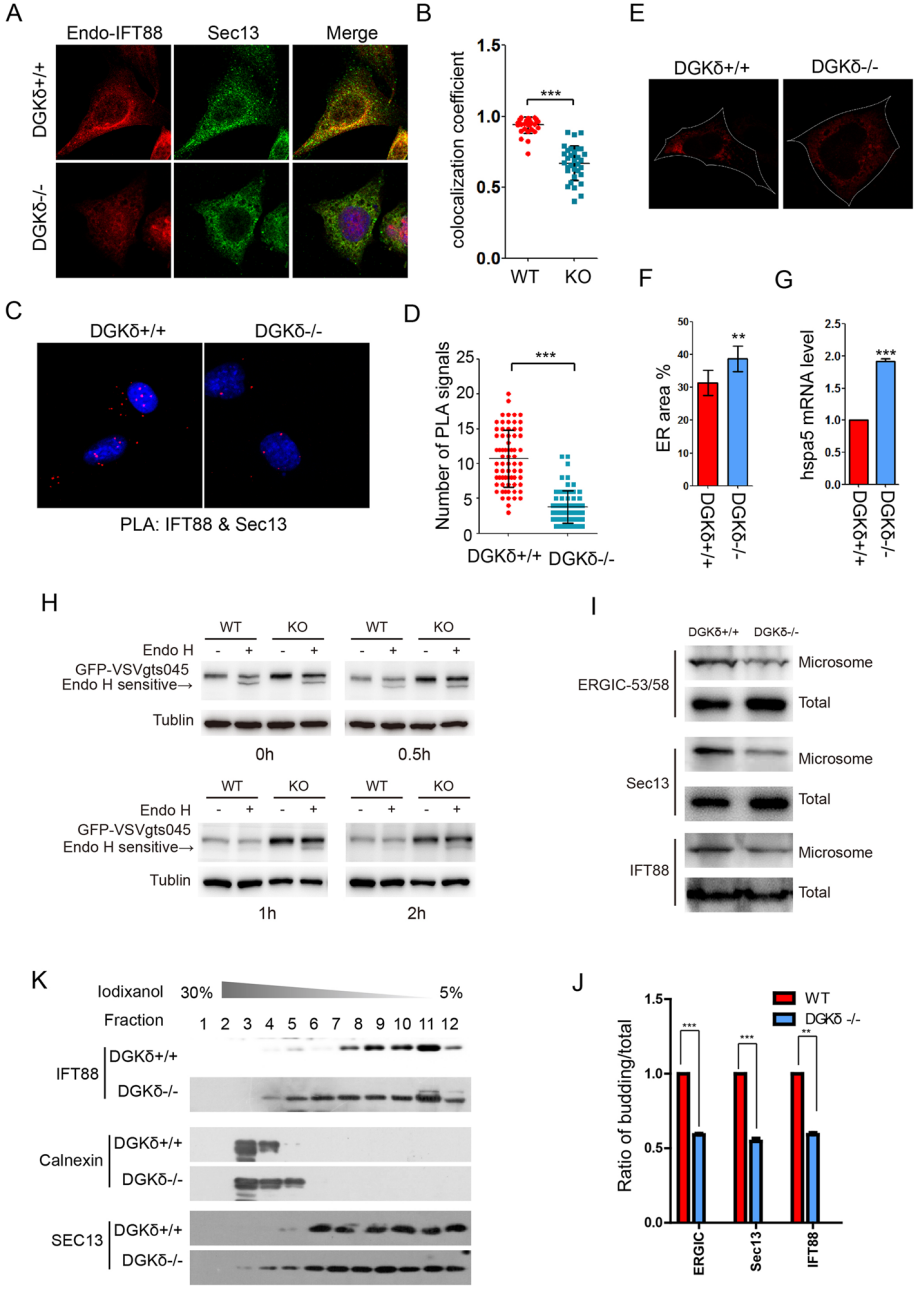
DGKδ triggers ER release of IFT88-containing COPII vesicles. (**A**) Representative immunostaining images of endogenous IFT88 and SEC13 in DGKδ^−/−^ and their matching control (DGKδ^+/+^) MEFs. (**B**) Calculation of colocalization co-efficient in (**A**). (**C**) Proximity ligation assay for and (**D**) quantification of the interaction between IFT88 and SEC13 in DGKδ ^−/−^ MEFs and the control (DGKδ^+/+^) MEFs. Data are means plus standard deviation and the statistical significance was calculated with the Student T test, n > 40. (**E**) Representative images and (**F**) quantification of ER areas in DGKδ^+/+^ and DGKδ^−/−^ MEFs. The cells were marked by exogenously expressed DsRed-ER, and the white dotted lines mark the contour of the cell. Data are means plus standard deviation and the statistical significance was calculated with the Student T test, n > 20. (**G**) Real-time qPCR analysis of Hspa5 mRNA in DGKδ^+/+^ and DGKδ^−/−^ MEFs. (**H**) Westernblot analyses Endo H sensitive of VSVg^ts045^ in DGKδ^+/+^ and DGKδ^−/−^ MEFs. The cells were routinely maintained at the permissive 37 °C, shifted to the nonpermissive 40 °C for 24 hours to let GFP-VSVg^ts045^ accumulate in the ER after transfection with a plasmid vector expressing GFP-VSVg^ts045^, then added cycloheximide to inhibit protein synthesis and shifted back to permissive 32 °C for various time periods to monitor VSVg^ts045^ trafficking after adding cycloheximide to inhibit protein synthesis. At the end of each time point, the cells were harvested for Endo H digestion and analyzed by westernblot. (**I**) Western blot results showing the distribution of ERGIC53, IFT88 and Sec13 in COP II-coated vesicles which budding *in vitro*. And the distribution of the three proteins and β-actin were taken as the control. (**J**) Bar graph depicts the ratio of relative intensity of ERGIC53, Sec13 and IFT88 in budding and total portions in DGKδ^+/+^ and DGKδ^−/−^ MEFs in (**I**). (**K**) Iodixanol density gradient sedimentation of calnexin, IFT88, SEC13, and exogenously expressed HA-DGKδ in DGKδ^+/+^ and DGKδ^−/−^ MEFs. **P < 0.01, ***P < 0.001.

To ascertain if DGKδ indeed plays a role in the assembly of COPII vesicles or their release from the ER, we compared the localization of endogenous IFT88 at COPII vesicles marked by SEC13 in DGKδ^−/−^ and matching wild type control MEFs. The results showed that in the absence of DGKδ, there is a 30% reduction of IFT88 presence in COPII vesicles, as evident from the calculation of the colocalization coefficient of IFT88 and SEC13 (Fig. 4A and B). Proximity ligation assay confirmed the reduction in the colocalization between IFT88 and SEC13 in DGKδ^−/−^ MEFs (Fig. 4C and D). Using DsRed-ER to decorate the ER, we found that the ER volume in DGKδ^−/−^ MEFs was significantly enlarged compared to that in the wild type controls (Fig. 4E and F), and moreover, the expression of Hspa5, a marker of ER stress^34^, also increased significantly (Fig. 4G). This observation was likely caused by an impairment of COPII vesicles-mediated export that rendered the ER swollen. To directly distinguish if the defect caused by the loss of DGKδ is associated with the assembly or the release of COPII vesicles, we conducted an ER transport assay in DGKδ^−/−^ and wild type control MEFs employing an temperature sensitive mutant, GFP-tagged VSVg^ts045^, which is retained in the ER when the cells are cultured at the non-permissive temperature of 40 °C, but moves out of the ER to Golgi apparatus at the permissive 32 °C^35^. Secretary proteins such as VSVg are glycosylated concurrent with their synthesis in the rough ER and thus are sensitive to digestion by Endoglycosylase H (Endo H) until they enter the Golgi apparatus, where the oligosaccharide side chains are modified^36^. Indeed, upon adding cycloheximide to inhibit protein synthesis and switching to permissive temperature of 32 °C, we found that GFP-VSVg^ts045^ began to gradually lose its sensitivity to Endo H until that sensitivity was completely lost within 2 hours of temperature shift in control wildtype MEFs, but the Endo H sensitivity persisted beyond this time frame in DGKδ^−/−^ MEFs (Fig. 4H), implying a blockage or at least a delay of ER export. Furthermore, we quantified the impact of DGKδ depletion on the COPII vesicle formation out of the ER membrane in a reconstituted *in vitro* “budding” system using the total membrane fractions isolated from DGKδ^−/−^ and wild type control MEFs supplemented with ATP-regenerating system and rat liver lysates^37^. After the budding reaction, we isolated the COPII vesicles of the microsome fraction and quantified the contents of Sec13, IFT88, and the ER-Golgi intermediate compartment (ERGIC) marker protein ERGIC53 enriched therein by Western blot. The results showed less COPII vesicle budding in the absence of DGKδ as evident by the reduced budding to total ratios of the three marker proteins (Fig. 4I and J). Finally, in the iodixanol density gradient equilibrium centrifugation experiment, while the majority of both IFT88 and SEC13 were associated with free COPII vesicles in normal MEFs, a significant fraction of these two proteins were retained in the ER in DGKδ^−/−^ MEFs (Fig. 4K). Taken together, these results suggest that DGKδ is required to trigger the release of IFT88-containing COPII vesicles from the ER.

### DGKδ is required for the assembly and function of primary cilia

**Figure 5:**
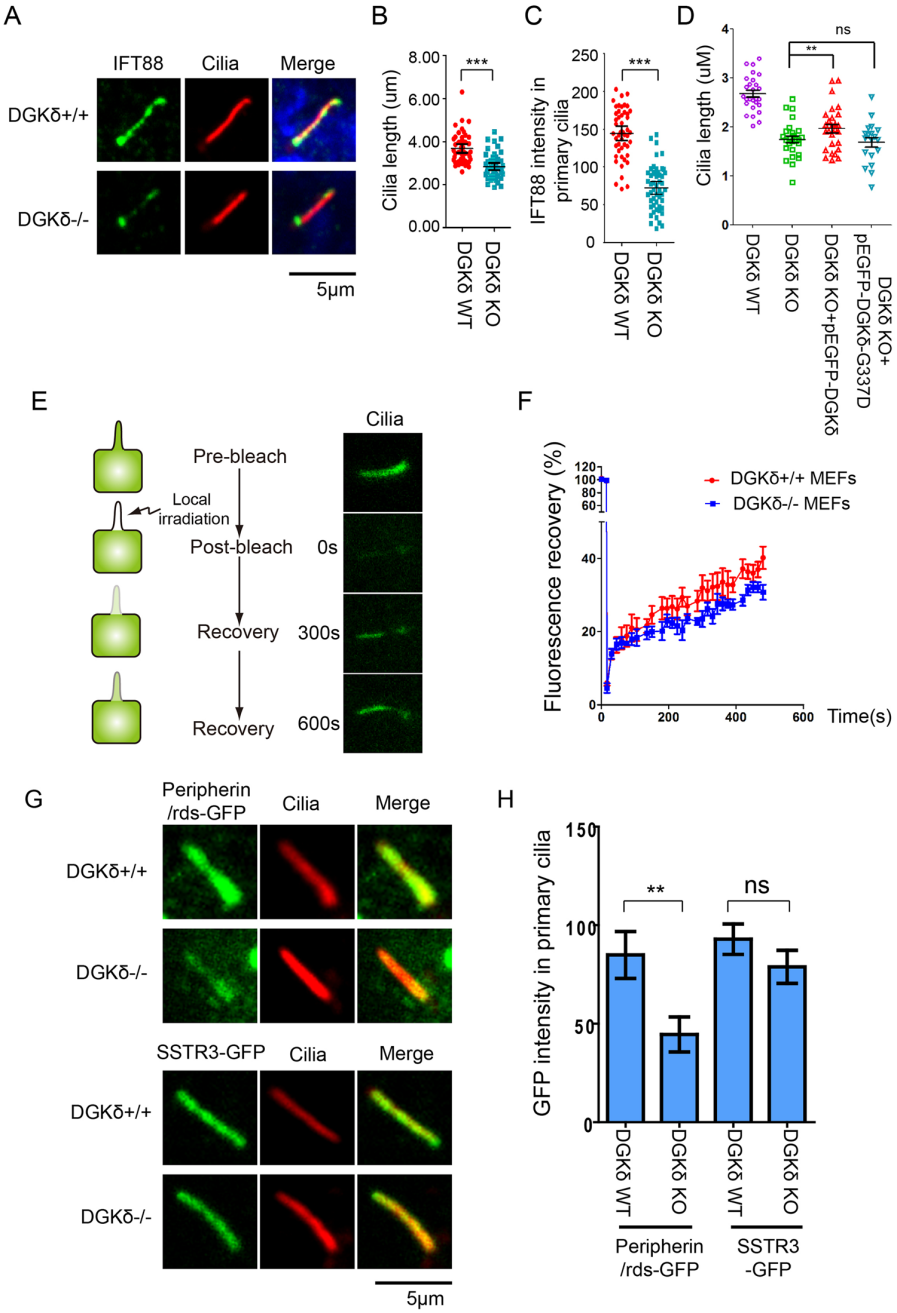
DGKδ is required for maintaining cilium length and IFT transport. (**A**) Immunofluorescence staining of IFT88(green) and acetylated tubulin (red) in DGKδ^+/+^ and DGKδ^−/−^ MEFs. The cells were allowed to reach confluency, then serum starved for 24 hours before being analyzed. (**B**) Quantification of cilium length and (**C**) intensity of IFT88 staining in (**A**) are presented as means ± SD, N > 20. (**D**) Quantification of cilium length in DGKδ^−/−^ MEFs transfected with pEGFP, pEGFP-DGKδ and pEGFP-DGKδG337D. The cells were serum-starved post-transfection for 24 hours to allow for cilium formation. N > 20. (**E–F**) FRAP measurement of IFT88-GFP import into the primary cilium in DGKδ^+/+^ and DGKδ^−/−^ MEFs. The cells were treated as in (**D**), and images of fluorescence recovery were captured every 30 s after photobleaching in the entire cilium. (**G**) Immunofluorescence measurement and (**H**) quantification of exogenously expressed GFP-Peripherin/rds and GFP-SSTR3 in primary cilia of DGKδ^+/+^ and DGKδ^−/−^ MEFs. The cells were stained with anti-Ac-tubulin to mark the axoneme of cilia. Data are means plus SD, and statistical analysis was carried out with Student’s T test. **P < 0.01, ***P < 0.001.

To determine if the DGKδ-mediated release of the IFT88-containing COPII vesicles affects cilium formation and function, we examined the physical properties of the primary cilium in WT and DGKδ^−/−^ MEFs. The results showed that in the absence of DGKδ, both the cilium length and the IFT88 content decreased significantly (Fig. 5A–C). To ascertain if these observations were consequential to the specific loss of DGKδ, we reintroduced the GFP-tagged, wildtype DGKδ or DGKδG337D, a mutant defective in kinase activity, into DGKδ^−/−^ MEFs and found that wildtype DGKδ was able to rescue the cilium length defect, but DGKδG337D was not (Fig. 5D). Because the total level of IFT88 protein remained comparable between DGKδ^−/−^ and the normal control MEFs (sFig. S3), the shortening of the cilium length could be the consequence of the reduced intraflagellar transport, which is known to be required for maintaining the integrity of the dynamic ciliary structure^38^. To test this explanation, we carried out fluorescence recovery after photobleaching (FRAP) measurement of the ciliary import of IFT88-GFP in DGKδ^−/−^ and normal MEFs by recording the accumulation of GFP fluorescence in the entire primary cilium every 30 seconds for 10 consecutive minutes after photobleaching (Fig. 5E). The results showed a clear reduction in the rate by which IFT88-GFP was reimported back into the primary cilium in DGKδ^−/−^ MEFs (Fig. 5F). These results indicated that loss of DGKδ indeed caused a curtailment of IFT88 trafficking into the primary cilium, hence the shortening of ciliary length.

Movement of IFT vesicles from the Golgi apparatus to the primary cilium has been reported in the literature^19, 39^. This is exemplified by the ciliary import of Somatostatin receptor R3 (SSTR3) that follows the conventional route of secretory pathway^40^. On the other hand, the incorporation of Peripherin/rds into the outer segment of photoreceptor cells follows an unconventional secretary route that bypasses the Golgi^41^. Since the outer segment of a photoreceptor is a specialized primary cilium, we compared the levels of GFP-Peripherin/rds and GFP-SSTR3 in the primary cilium of DGKδ^−/−^ MEFs to assess the requirement of DGKδ by these two alternative transport routes. Our data showed a robust presence of GFP-Peripherin/rds in the primary cilia of normal MEFs as expected, but its level in DGKδ^−/−^ MEFs was reduced considerably (Fig. 5G and H). In contrast, the levels of GFP-SSTR3 in the primary cilium were comparable in both cell types (Fig. 5G and H). These results strongly support the notion that DGKδ controls the ER release of IFT vesicles destined to the primary cilium.

### DGKδ is required for Shh signaling

**Figure 6:**
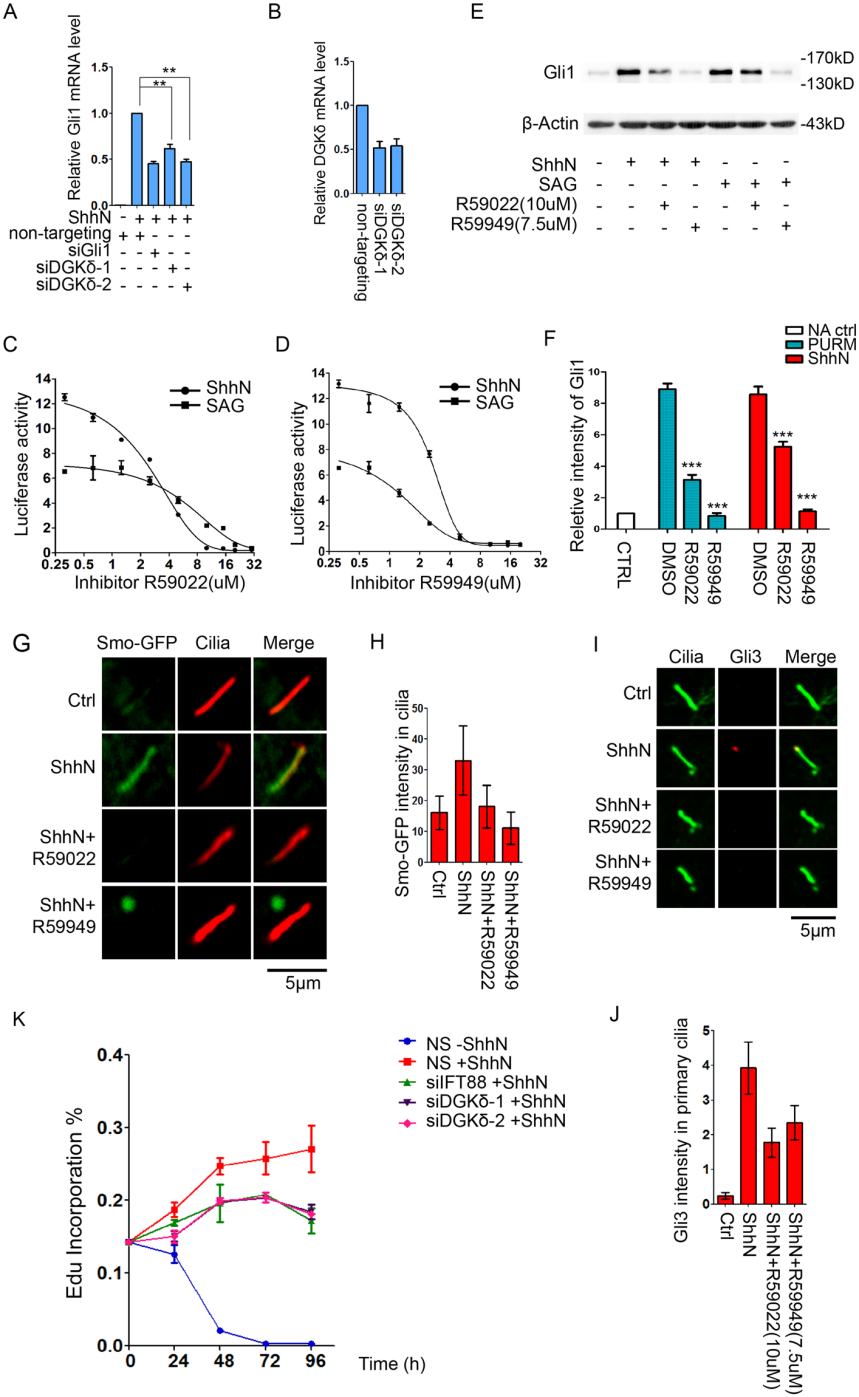
DGKδ is required for supporting Shh signaling functions. (**A**) Real-time qPCR analyses of Gli1 mRNA in NIH3T3 transfected with various siRNAs as indicated. ShhN-CM (1:10) was given for 12 hours. (**B**) Real-time PCR analyses of siDGKδ-1 and siDGKδ-2 knockdown efficiency in NIH3T3. (**C**,**D**) 8xGBS-luc reporter assays for the ability of DGK inhibitor R59022 (**C**) and R59949 (**D**) to inhibit Shh signaling induced by ShhN-CM (1:10) or SAG. (**E**) Western analysis of Gli1 protein level induced by ShhN or SAG in the absence or presence of R59022 (10 μM) or R59949 (7.5 μM). (**F**) Quantification of Gli1 intensity in (**E**). (**G**) Representative confocal images of Smo-GFP in cilia of Smo^−/−^ MEFs, in which Smo-GFP was re-introduced by stable transfection. The treatment with ShhN-CM and R59022 or R59949 was given for 4 hours. (**H**) Quantification of Smo-GFP intensity in (**G**), N > 20. (**I**) Representative images of immunofluorescence staining and (**J**) quantification of Gli3 in cilia of NIH3T3 cells. The treatment was given as in (**G**). (**K**) Quantification of EdU incorporation by freshly isolated cerebellar GCP cells. The cells were transfected with non-targeting siIFT88, siDGKδ-1, siDGKδ-2, or a non-silencing control (NS), and treated with or without ShhN as indicated.

To assess the functional significance of the role of DGKδ, we carried out a number of loss-of-function studies of Shh signaling. First, siRNA-mediated knockdown of DGKδ blunted the ability of Shh to induce the expression of Gli1, a well-known pathway target^42^ (Fig. 6A and B). Second, pharmacological intervention with two pan-DGK inhibitors, R59022 and R59949, showed specific inhibition of the Shh pathway activation assessed by the 8xGBS-luc reporter assay^12^ (Fig. 6C and D), and Western analysis of endogenous Gli1 protein levels (Fig. 6E and F). Interestingly, the pathway activation by the ligand (ShhN conditioned medium) showed much higher sensitivity to these two compounds than that by SAG, an agonist of the Shh pathway signal transducer Smo^43^ (Fig. 6C and D). Third, we reconstituted Smo^−/−^ MEFs with a GFP-Smo expression construct in an attempt at generating a marked Smo stable cell line with Shh responsiveness. These cells exhibited about 2-fold increase in GFP-Smo fluorescence intensity in the primary cilium upon ShhN induction (Fig. 6G and H); however, treatment with DGK inhibitors blocked such induction. Moreover, blocking DGKδ kinase activity with the inhibitors also reduced the ciliary accumulation of endogenous Gli3 induced by ShhN (Fig. 6I and J), another established Shh signaling response^10^. Finally, we isolated cerebellar granule cell precursors (GCPs) from developing 7 days old pups, and cultured them *in vitro*. These cells absolutely depend on Shh for sustaining their survival and growth in culture^44^. When we knocked down the expression of DGKδ using siRNAs, the growth of GCPs was substantially compromised to the same extent as knockdown of IFT88 (Fig. 6K). Taken together, both reduction of DGKδ’s expression or blocking its kinase activity negatively affected Shh signaling, demonstrating a requirement of this kinase activity.

## Discussion

Although characterization of *flexo/Polaris/Tg737* mutants led to the first insight of the primary cilium as the venue of Shh signaling over a decade ago^3^, the precise function of IFT88 was still not clear. In an attempt at identifying direct link between IFT88 and components of the Shh pathway, we serendipitously discovered DGKδ as a novel binding partner of IFT88. Our data indicate that IFT88 is present in the COPII-coated vesicles and DGKδ plays a critical role in releasing these vesicles from the ER, thus setting forth their movement into the primary cilium. Since the biosynthetic machinery is not present inside cilia, transport by IFT88-containing vesicles is essential not only for the delivery of protein and lipid cargos, but also the maintenance of ciliary structure and function required for supporting Shh signaling.

Despite the name-sake, IFT proteins share sequence homology with coat proteins of intracellular vesicle and have been implicated in transport underlying exocytosis^20, 39, 45^. For instance, IFT20 was found in post-Golgi membrane compartments and has been shown to guide vesicles transport between the Golgi apparatus and cilium basal body as well as centrosomes in both ciliated and non-ciliated cells^22, 46^. During the formation of immune synapses, IFT88 was reported to be associated with IFT20 and IFT57 in the recycling endosomes^22^. In a study of USHER syndrome, IFT88 was reported to form a complex with Cdh23, Harmonin and Myo7aa to mediate the trafficking of USH proteins from the ER to the ER-Golgi intermediate compartment (ERGIC)^23^. Our data clearly showed that IFT88 is associated with the ER as well as ER-derived COPII vesicles (Figs 1A–D and 4I), and colocalization with SEC16A (Fig. 3D), a marker of ERES, strongly suggests that IFT88 is likely incorporated into COP II vesicles at the ER exit sites. Furthermore, our data also indicated that the DGKδ-mediated release of IFT88-containing COPII vesicles from ERES plays a critical role in the maintenance of cilium length and function, despite its site of action situates several steps upstream from cilia, implying a coordinated control between the biosynthesis activity at the ER and the assembly and function of the primary cilium.

**Figure 7:**
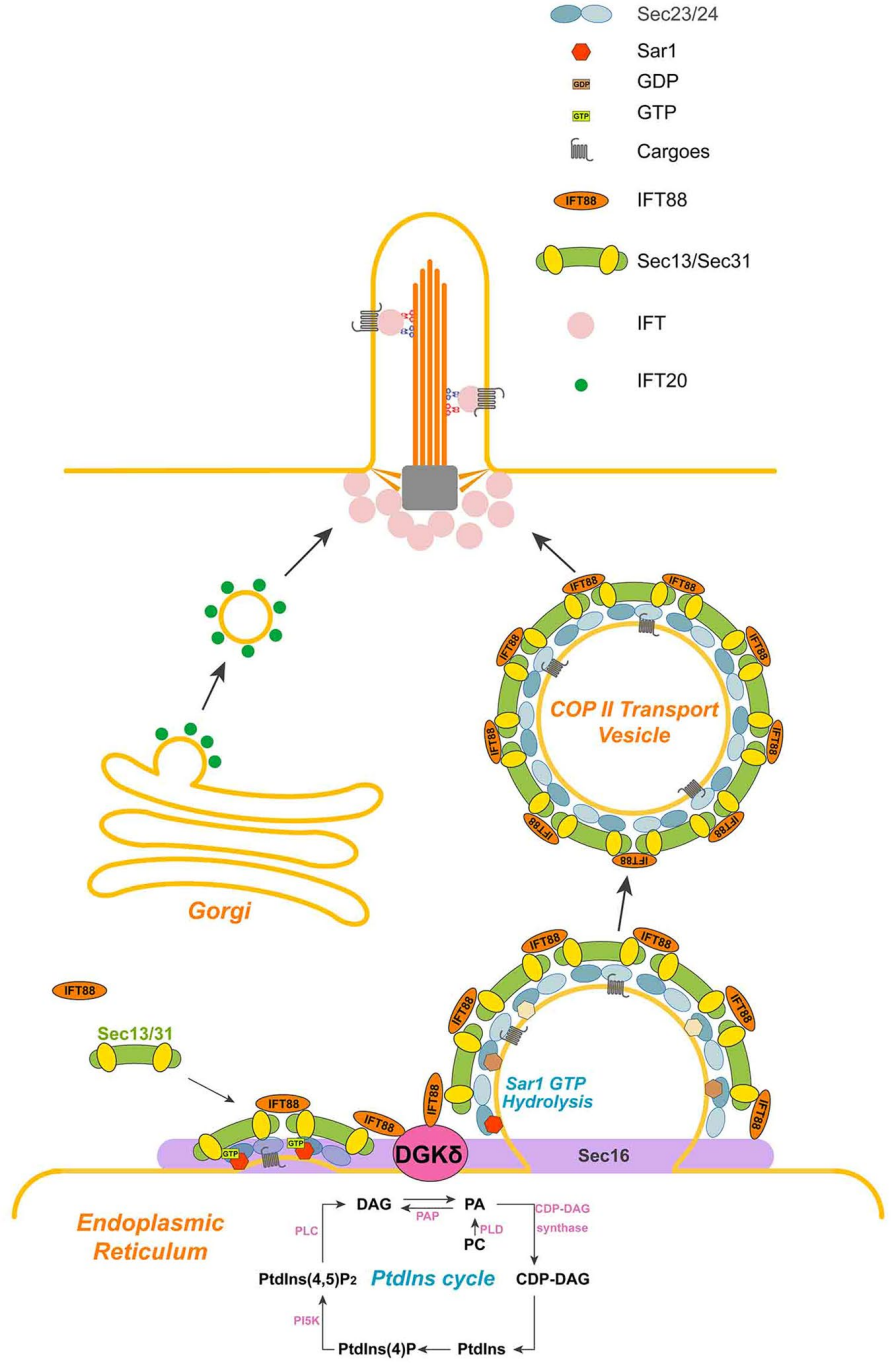
A model for IFT88 mediated secretory pathway from ER to primary cilia. IFT88 is found at the ER exit sites, where it is incorporated into the ER-derived COP II-coated vesicles. The IFT88 containing COP II vesicles travel directly to cilia bypassing the Golgi apparatus. The newly identified IFT88-binding protein DGKδ is colocalized with IFT88 at the ER exit sites and is required for triggering the release of IFT88 vesicles from the ER.

Small GTPases-driven vesicular transport is known to be the main route that delivers various protein and lipid cargos from their site of synthesis in the cell body to the primary cilium^47^, but recent data have implicated IFT20 in transporting cargos from the Golgi or post-Golgi membrane compartments to the primary cilium as well^22, 46, 48^. Our finding of IFT88-containing COPII vesicles revealed another important pathway for supplying cilium assembly and function. Despite repeated effort, we did not find IFT88 associated with any Golgi marker, suggesting the IFT88-mediated transport is completely separate from that of IFT20. It is conceivable that these two different transporting systems play complementary roles in delivering their respectively unique types of cargos, and are subject to different types of controls (Fig. 7). Recent data also revealed that certain transmembrane receptors, such as cilium-targeted peripherin in the retina, follow unconventional secretory pathways traversing directly from the ER to the plasma membrane, bypassing the Golgi^41^. A direct ER to cilia transport route could ensure a timely delivery of cargos to the plasma membrane or alternatively preserve protein activities by avoiding unnecessary processing in the Golgi. In this regard, the IFT88-mediated transport of peripherin/rds to the primary cilium offers a good example.

Out of the ten DGKs encoded in the mammalian genomes, only DGKδ and DGKε have been reported in the ER^25, 49^. Although one study showed that overexpressing DGKδ caused a decrease in the ER export^25^, the precise function of this lipid kinase is not clear. We show here that the DGKδ is associated with Sec16 at the ER exit site and its kinase activity is required for releasing IFT88/Sec13 vesicles, thus triggering ER export. Besides protein synthesis, ER is also the site for synthesis of phospholipids, the composition of which in the ciliary membrane is very important for its function^50, 51^. We can speculate that IFT88-containing vesicles might supply the primary cilium membrane with unique phospholipid composition as these vesicles fuse with the basal body. Using a combination of molecule inhibitors, siRNA-mediated gene silencing as well as gene knockout approaches, we demonstrated that Shh signaling can be strongly inhibited both *in vitro* and *in vivo* by the blockade of DGKδ kinase activity, despite the functional redundancy. Thus, these DGK blocking compounds or their derivatives offer a new way of treating cancers derived from inappropriate Shh signaling.

## Materials and Methods

### Cells and plasmids

HEK293 and NIH3T3 cells were purchased from ATCC, and DGKδ^−/−^ MEFs were provided to us as a generous gift from Dr. Matthew K. Topham^28^. Gli-Luc 3T3 cells were obtained from StemRD. Smo-GFP stable cells were derived from Smo^−/−^ MEFs by stable transfection. PCMV-IFT88-3XFLAG expression vector was obtained from Dr. Gregory J. Pazour, and pCMV5-HA-hDGKα, β, γ, δ, ε, ζ, ι vectors were obtained from Dr. Robert J. Lefkowitz. IFT88 deletion constructs (ΔTPR1, ΔTPR2, ΔTPR1&2, Δ293~400) were generated by PCR from pCMV-IFT88-3XFLAG and subcloned into the pRK1F vector. GFP-IFT88 was subcloned in pEGFPN1. DGKδ deletion constructs (N-terminal, catalytic domain, and C-terminal) were generated by PCR and subcloned into pRK2H. SEC13 cDNA was generated from NIH3T3 total RNA and the GFP- SEC13 expression unit was subcloned in pEGFPN1. GFP-Sec16A and EGFP-VSVG plasmids were obtained from Addgene. DsRed-ER, CFP-Gal, CFP-TGN38 constructs were provided by Dr. Li Yu (Tsinghua University, China).

### siRNA mediated knockdown and real-time qPCR

Target sequences of DGKδ-specific siRNAs are siDGKd-1, AAGACUUGUGGCAGCGUGUUA and siDGKd-2, CAGCUCCAUCAUUCGAUGAUA. Both siRNAs were purchased from Qiagen (Gaithersburg, MD). The target sequence of IFT88-specific siRNA is: ACUGGGAGAGUUAUACGAU, which was purchased from Ribo, Ltd (China). Levels of mRNAs were determined by real-time qPCR using the FastStart SYBR Green Master mix (Roche) on a 7500 Real-Time PCR System (Applied Biosystems). The sequences of PCR primer pairs are: mouse DGKδ (GCCGACAACAGGAAAGAAATG, TTGGGTAGGCTCAAAATGCTC), mouse Gli1 (GCTTGGATGAAGGACCTTGTG, GCTGATCCAGCCTAAGGTTCTC), and mouse HPRT (TATGGACAGGACTGAAAGAC, TAATCCAGCAGGTCAGCAAA), mouse hspa5 (CTGGACTGAATGTCATGAGGATCA, CTCTTATCCAGGCCATATGCAATAG). In each experiment, measurements were repeated at least three times, and each data point consists of triplicate samples.

### Immunoflurescence

NIH3T3 or MEFs were seeded on glasses and cultured for 24 hours prior to transfection with Fugene HD (Promega). The cells were allowed to recover for 24 to 36 hours. To visualize ciliary proteins, transfected cells were serum-starved in DMEM containing 0.5% FBS for 24 hours before other treatments. Fixation was done in 4% paraformaldehyde for 10 minutes at room temperature, permeablized with 0.3% Triton X-100 in PBS for another 10 minutes, followed by standard procedures for immunostaining. Primary antibodies used were rabbit anti-IFT88 (1:100; Proteintech), goat anti-SEC13 (1:50; Santa Cruz Biotechnology, Inc.), rabbit anti-Calnexin (1:100, Proteintech), rabbit anti-Clathrin heavy chain (1:200; Cell Signaling Technology (Danvers, MA)), rat anti-HA (1:300; Roche), mouse anti-acetylated Tubulin (1:1000; Sigma), rabbit anti-Gli3 (1:500; R&D (Minneapolis, MN)). Alexa-coupled secondary antibodies were purchased from Life Technologies Corp. Fluorescence images were captured using a laser scanning confocal microscope (LSM 710; Carl Zeiss) with a 63 × 1.4 numerical aperture (NA) oil objective and images were analyzed using software Zeiss ZEN 2011. Colocalization was quantified by software Image-pro as described^52^.

### Cell fractionation

NIH3T3 cells were homogenized in 0.25M sucrose, 10 mM Tris-HCl, pH7.4, plus 1x Roche cOmplete Protease Inhibitor Cocktail, and cleared by centrifugation at 800 g at 4 °C for 10 minutes. Mitochondria were pelleted by centrifugation at 10,000 g at 4 °C for 20 minutes. The supernatant fraction was centrifuged again for 4 hours at 100,000 g to isolate cytosolic and microsome fractions.

### Mass spectrometry analysis of IFT88 binding proteins

Endogenous IFT88 in NIH3T3 cell lysates was immunoprecipitated with anti-IFT88 (Proteintech), resolved by 10% SDS-PAGE gel and silver-stained. The unique bands in the anti-IFT88 lane were from excised and sent for LC/MS-MS analysis.

### Immunoprecipitation

The cells were lysed in RIPA (50 mM Tris-HCl pH 7.4, 150 mM NaCl, 1 mM EDTA pH 8.0, 1% NP-40, 0.5% Sodium Deoxycholate, and 1 x Roche cOmplete Protease Inhibitor Cocktail). For figure2A, NIH3T3 cell lysates (500 ul, 1 mg/ml) were precipitated with 2 ul normal IgG control or anti-IFT88 (4 °C overnight with protein A agarose beads) and subjected to PAGE resolving and silver staining. For the others, desired proteins (500 ml, 1 mg/ml) were immunopurified with 2 ul anti-Flag M2 agarose beads (Sigma) or 2 ul anti-HA (Roche) with protein G agarose beads (Millipore) as indicated. The isolated proteins in the pellet were analyzed by Western analysis.

### Proximity ligation assay

Cells were cultured, transfected and stained with primary antibodies as described under the Immunofluorescence. The cells were then incubated with secondary antibodies conjugated with oligonucleotides (PLA probe MINUS and PLA probe PLUS) in Duolink^®^ *In Situ* PLA^®^ Probe reagents (Sigma). The oligonucleotides ligation, amplification and fluorescently labeling were performed with Duolink^®^ *In Situ* detection reagent (Sigma). The images were captured on a Carl Zeiss LSM710 microscope using a 63×, 1.4 numerical aperture (NA) oil objective.

### OptiPrep™ Density gradient sedimentation

Iodixanol density gradient sedimentation was carried as described^53^. Briefly, NIH3T3 or MEF cells were homogenized in 0.25 M sucrose, 10 mM Tris-HCl, pH 7.4, 1× Roche cOmplete Protease Inhibitor Cocktail and centrifuged at 800 g at 4 °C for 10 minutes to isolate postnuclear supernatant. OptiPrep gradient of 5–30% (5% increment and 1.5 ml for each gradient step) was placed in ultracentrifuge tubes (Beckman), and 3 ml of postnuclear supernatant was overlaid on top of the gradient. After centrifugation at 40,000 rpm for 4 hours at 4 °C in an SW 41 Ti rotor on Beckman Optima L-100 XP ultracentrifuge, the gradient was collected in 12 equal volume fractions using a peristaltic pump from the bottom of the tube. Proteins in each fraction were then analyzed by Western blot.

### Live cell imaging and FRAP

DGKδ^+/+^ and DGKδ^−/−^ MEFs were seeded in Lab-Tek chambered slides and transfected with GFP-IFT88. After reaching confluency, the cells were serum-starved in DMEM containing 0.5% FBS for 24 hours to induce cilia. To capture live cell images, the cells were incubated in CO_2_-independent medium (Life Technologies) and placed on a heating stage pre-warmed to 37 °C during images capture using Carl Zeiss LSM710 microscope live cell confocal imaging work station. Primary cilia were identified in the 488nm channel based on IFT88-GFP fluorescence. Photobleaching experiments were carried out using the FRAP module of the Marianas system. Equivalent laser intensity, repetition, and exposure time were used for FRAP experiments. Bleached regions were accurately positioned along each cilium at a 30-second interval for 10 consecutive minutes.

### VSVg Endo H sensitivity

DGKδ^+/+^ and DGKδ^−/−^ MEFs were transfected with GFP-VSVg^ts045^ plasmid using Lipofectamine and PLUS (Invitrogen). Immediately after transfection, the cells were incubated at 40 °C to allow GFP-VSVg^ts045^ protein to accumulate in the ER. Twenty-four hours after incubation at 40 °C, the cells were transferred to the permissive temperature at 32 °C for the appropriate time period (0 h, 0.5 h, 1 h, 2 h). Cells were then transferred to ice, and washed twice with ice-cold PBS and lysed for protein collection. After protein concentration determination using the Bradford assay (Bio-Rad, Hercules, CA, USA), equal amounts of protein from all samples were treated or mocktreated with Endoglycosidase H (Roche, Indianapolis, IN, USA) according to the manufacturer’s instructions. Samples were subjected to western blot analysis using anti-GFP (Sigma) and anti-actin antibodies.

### Cell free COPII budding assay

DGKδ^−/−^ and matching wildtype control MEFs grown to 70–80% confluency were washed once with PBS, scraped into PBS (5 ml/dish), pooled, and harvested at 1000 × g for 5 min at 4 °C. The pellet was resuspended in PBS (0.5 ml/dish) and centrifuged again at 1000 × g for 5 min at 4 °C. The pellet was then resuspended in Buffer F (10 mm HEPES-KOH (pH 7.2), 250 mm sorbitol, 10 mm potassium acetate, 1.5 mm magnesium acetate plus protease inhibitor mixture), passed through a 22-gauge needle 20 times, and centrifuged at 1000 × g for 5 min at 4 °C. The post nuclear supernatant fraction was transfered into siliconized microcentrifuge tubes, and microsomes were sedimented at 6000 × g for 10 min at 4 °C. Harvested microsomes were washed twice with Buffer G (20 mm HEPES-KOH (pH 7.2), 250 mm sorbitol, 150 mm potassium acetate, 0.5 mm magnesium acetate plus protease inhibitor mixture, and the pellet was resuspended in Buffer G (30 μl/dish). 30 ul of microsomes was used for each *in vitro* vesicle-formation reaction. Equal amount of protein in the microsome fraction was used in each assay. The budding reaction was assembled using rat liver cytosol (Invitrogen) at 4 mg/ml and an ATP-regenerating system (Boston Biochem, 1:10) on ice and then incubated for 30 min at 30 °C. The donor membrane were removed by a 20-min 12,000 × g centrifugation, and the supernatant fraction was further centrifuged for 30 min at 55,000 rpm at 4 °C using a TLA100 rotor in a Beckman Optima TLX ultracentrifuge to sediment the vesicle products. At last the samples were subjected to western blot analysis with anti-IFT88, anti-Sec13, anti-ERGIC53/58.

### GCP isolation and proliferation assay

Mouse cerebellar GCPs isolation assay were performed as described^54^. Cerebella were dissected out from 6-day-old pups, incubated with Trypsin/DNAse buffer at 37 °C for 5 minutes and squeezed through a fine Pasteur pipette to obtain single-cell suspension. To separate different types of cells, the suspension was overlaid on top of 35% and 60% Percoll step gradient and centrifuged at 2,000 g for 10 minutes at 4 °C. GCP cells were harvested from the 60% percoll gradient fraction and further purified by depleting adherent cells with two consecutive 1-hour incubations in tissue culture dishes. For EdU(5-ethynyl-2′-deoxyuridine) incorporation assay, purified GCP cells were seeded in 24-well plates pre-coated with poly D-lysine and Matrigel solution, and incubate at 37 °C, 5% CO_2_. After 1 hour, GCP cells were transfected with siRNAs using FugeneHD Transfection Reagent (Promega) in the plates and the proliferation of GCPs at different time points was detected using Click-iT EdU cell proliferation assays (Life Technologies).

## Electronic supplementary material

Supplementary Information
