# Supplementary material for: DGKδ triggers endoplasmic reticulum release of IFT88-containing vesicles destined for the assembly of primary cilia
Source: Sci Rep. 2017 Jul 13;7:5296. doi: 10.1038/s41598-017-05680-8 (PMC5509727; doi:10.1038/s41598-017-05680-8)
Supplement: Supplementary file 1 — Supplementary Information [file 41598_2017_5680_MOESM1_ESM.pdf]

DGKδ triggers endoplasmic reticulum release of IFT88-containing vesicles destined for the assembly of primary cilia

Jie Ding, Lei Shao, Yixing Yao, Xin Tong, Huaize Liu, Shen Yue, Lu Xie and Steven Y Cheng[1]

Department of Developmental Genetics, School of Basic Medical Sciences, Nanjing Medical University, Nanjing, Jiangsu 211166

¶ Correspondence and material requests should be addressed to sycheng@njmu.edu.cn

SUPPLEMENTARY INFORMATION

Key words: Sonic hedgehog, primary cilium, vesicular transport, IFT88, and diacylglycerol kinase

Running title: DKGδ triggers the release of IFT88 particles from ER

Supplement Figure Legends

Figure S1 IFT88 did not localize at golgi, endosomes or Clathrin-coated vesicles. Representative immunofluorescence images of endogenous IFT88 and markers of Golgi (CFP-Gal (**A**), CFP-TGN38 (**B**)), endosomes (Lamp1 (**D**), Rab5 (**E**), Rab7 (**F**)) or Clathrin-coated vesicles (antibody against Clathrin heavy chain) (**C**) in NIH3T3 cells respectively. IFT88 staining did not show obvious overlap with theses markers. (**G**) Representative immunofluorescence confocal images showing colocalization of exogenous CFP-Sec31A with endogenous IFT88. MOC (Manders Overlap Coefficient): 0.144±0.048 in (**A**), 0.29±0.097 in (**B**), 0.24±0.083 in (**C**), 0.33±0.068 in (**D**), 0.17±0.065 in (**E**), 0.26±0.038 in (**F**), 0.63±0.035 in (**G**)

Figure S2 DGKδ does not localized at primary cilia. Exogenously expressed GFP-DGKδ and the staining of endogenous IFT88 (red) showing that DGKδ colocalized with IFT88 at peri-nuclear region but not primary cilia. Primary cilia was indicated by the staining of acetylated alpha-tubulin (purple).

Figure S3 DGKδ$^{-/-}$ MEFs have comparable amounts of endogenous IFT88 as the normal control. Western blot of the total level of endogenous IFT88 protein between DGKδ$^{-/-}$ and the normal control MEFs indicated that there is no significant difference between these two cell lines on the expression level of IFT88.

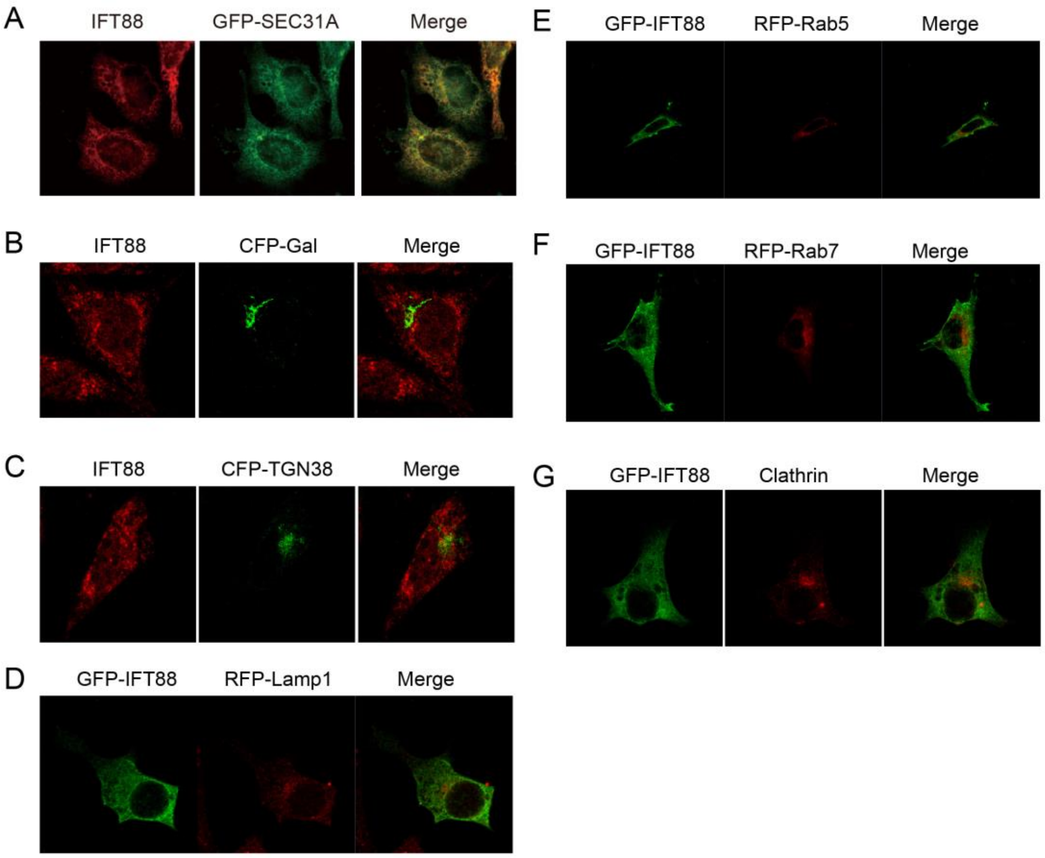
A
IFT88
GFP-SEC31A
Merge
B
IFT88
CFP-Gal
Merge
C
IFT88
CFP-TGN38
Merge
D
GFP-IFT88
RFP-Lamp1
Merge
E
GFP-IFT88
RFP-Rab5
Merge
F
GFP-IFT88
RFP-Rab7
Merge
G
GFP-IFT88
Clathrin
Merge

Figure S1

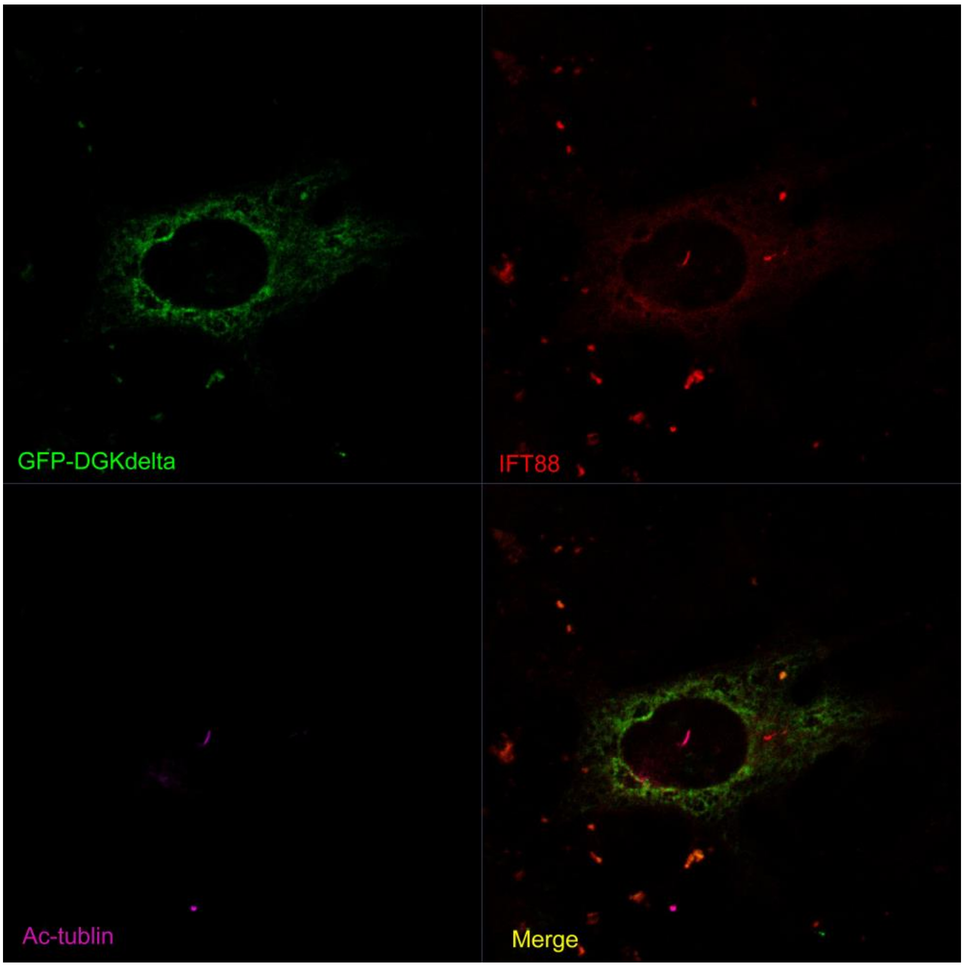

Figure S2

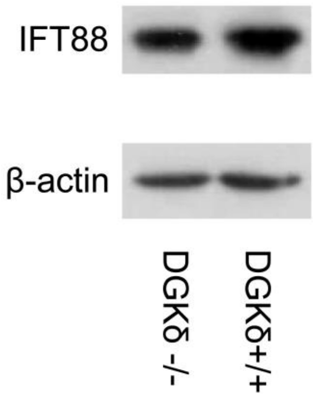

Figure S3
